# Patients’ Experiences of Telemedicine for Their Skin Problems: Qualitative Study

**DOI:** 10.2196/24956

**Published:** 2022-02-22

**Authors:** Aloysius Chow, Sok Huang Teo, Jing Wen Kong, Simon Lee, Yee Kiat Heng, Maurice van Steensel, Helen Smith

**Affiliations:** 1 Family Medicine and Primary Care Lee Kong Chian School of Medicine Nanyang Technological University Singapore Singapore; 2 National Healthcare Group Polyclinics Singapore Singapore; 3 National Skin Centre Singapore Singapore; 4 Skin Research Institute of Singapore Singapore Singapore; 5 Dermatology and Skin Biology Lee Kong Chian School of Medicine Nanyang Technological University Singapore Singapore

**Keywords:** teledermatology, qualitative, patients experience, telemedicine, dermatology, Singapore

## Abstract

**Background:**

Teledermatology is a cost-effective treatment modality for the management of skin disorders. Most evaluations use quantitative data, and far less is understood about the patients’ experience.

**Objective:**

This qualitative study aimed to explore patients’ perceptions of a teledermatology service linking public primary care clinics to the national specialist dermatology clinic in Singapore. A better understanding of patients’ experiences can help refine and develop the care provided.

**Methods:**

Semistructured in-depth interviews were conducted with patients who had been referred to the teledermatology service. The interviews were digitally recorded and transcribed before undergoing thematic content analysis.

**Results:**

A total of 21 patients aged between 22 and 72 years were recruited. The following 3 themes were identified from the data of patients’ experiences: positive perceptions of teledermatology, concerns about teledermatology, and ideas for improving the teledermatology service. The patients found the teledermatology service convenient, saving them time and expense and liberating them from the stresses incurred when making an in-person visit to a specialist facility. They valued the confidence and reassurance they gained from having a dermatologist involved in deciding their management. The patients’ concern included data security and the quality of the images shared. Nonetheless, they were keen to see the service expanded beyond the polyclinics. Their experiences and perceptions will inform future service refinement and development.

**Conclusions:**

This narrative exploration of users’ experiences of teledermatology produced rich data enabling a better understanding of the patients’ journey, the way they understand and interpret their experiences, and ideas for service refinement. Telemedicine reduces traveling and enables safe distancing, factors that are much needed during pandemics.

## Introduction

The Global Burden of Disease lists skin disease in the top 20 leading causes of disability-adjusted life years, and the 4th leading cause of disability worldwide [1]. With dermatological disorders being so prevalent, it is not surprising that many consultations with a primary care physician focus on skin symptoms. A study in the Netherlands reported that about 13% of patients visiting a primary care practice were seeking help for a skin problem [2], and in the UK, the estimate was even higher at 24% [3]. When there is diagnostic uncertainty or unresponsiveness to treatment, the primary care practitioner will need to refer the patient for an expert dermatological opinion.

When telemedicine was developing in the 1990s, dermatology was identified as one of the clinical areas that could readily benefit from this mode of practice as it is a very visual specialty. Its applicability to rural areas where specialist care is not readily available was noted [4]. Since then, teledermatology has been initiated widely, aided by advancements in technology and internet availability. There are 3 modes of teledermatology consultation, which are “store-and-forward,” live videoconferencing, and a combination of both. The store-and-forward teledermatology consultation involves digital images being sent to the expert for later review, whereas live videoconferencing consultations are synchronous, with the patient and the clinicians interacting in real time. The store-and-forward mode is less resource intensive and flexible and is thus more widely used in dermatology. When compared to conventional care or live videoconferencing, the store-and-forward mode costs less and reduces the disruption in the daily workflow of clinicians [5]. The store-and-forward mode also offers greater privacy for patients [6] but is disadvantaged by the lack of opportunity for the specialist to interact with the patient or ask for further images.

Teledermatology has been reported to be efficacious across different patient populations [7]. In Singapore, it has been used previously to manage skin problems in nursing home residents where the nurses or nurse aides photographed the lesions and uploaded these images for dermatological opinion [8]. The system was used regularly for diagnosis and follow-up and enabled residents to obtain dermatological care from the comfort of their residence. Preparation of the referral request was onerous, taking an average of 86 minutes of nursing time, but entailed less disruption and inconvenience than accompanying the resident to an outpatient appointment.

**Figure 1:**
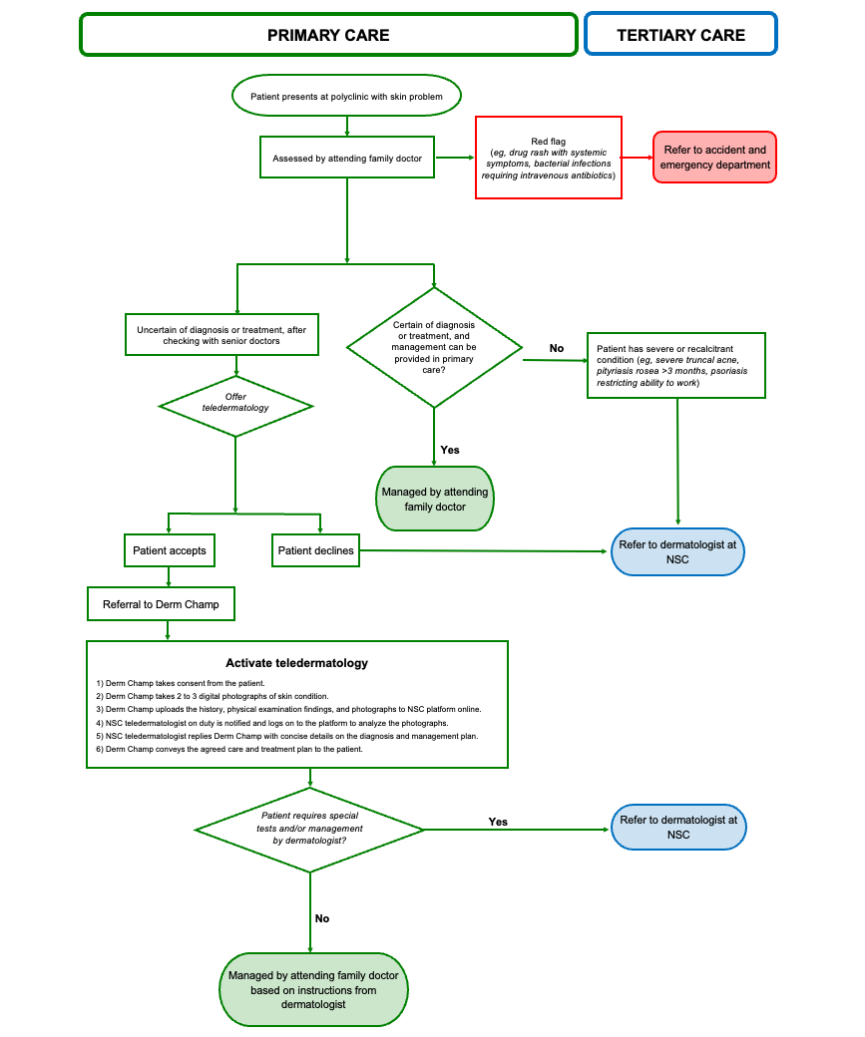
The TeleDERM process. NSC: National Skin Centre.

The National Healthcare Group Polyclinics are public primary care health facilities serving the central and northern parts of Singapore with an approximately 2.5 million attendances each year. Disorders of the skin and subcutaneous tissue are among the 10 most common diagnoses, with 45,987 in 2019 [9]. Traditionally, if the attending physician required advice on diagnosis and management, patients were referred to the National Skin Centre, a tertiary health care institution. With the aim of bringing specialist care closer to patients in order to reduce the expenditure and waiting time for specialist referrals and to increase the dermatology skills of family physicians, National Healthcare Group Polyclinics and National Skin Centre collaborated to introduce the first teledermatology service in primary care for Singapore [10]. Adopting the store-and-forward methodology, the clinical history and digital photographs of eligible patients are shared through a secure web portal and their management guided by a dermatologist without the need for a dermatological outpatient consultation [11] (Figure 1). This teledermatology process is mediated by “Derm Champs,” family physicians with a special interest in Dermatology and with a graduate diploma in Family Practice Dermatology or master’s degree in Family Medicine.

This study was designed to better understand the experiences of adult patients who had used the teledermatology services and to identify areas where their experiences could be improved.

## Methods

### Recruitment

The participants were attendees at one of 5 polyclinics, were English speaking, were at least 21 years old, and had undergone teledermatology within the last year. Eligible patients were identified by the medical staff involved in the telemedicine service within the polyclinics and at the National Skin Centre. The patients were invited to participate in this study when they attended for a follow-up consultation. They were given a leaflet about the study to consider at their leisure, and those willing to participate subsequently contacted the research team by mail, email, or telephone. This recruitment strategy was simple and was not resource intensive, but was not purposeful, and the 20 Singapore dollars (US $15) given as a token of appreciation may have encouraged respondents motivated by financial benefit.

### Data Collection

The face-to-face, semistructured interviews were conducted by a researcher trained in qualitative interview techniques. The topic guide explored patients’ experiences of the teledermatology service and how it could be improved for others (Multimedia Appendix 1). Interviews were audio recorded with the patient’s consent. One patient preferred not to be recorded, and the researcher took contemporaneous notes instead.

### Data Processing and Analysis

Digital audio recordings were transcribed verbatim. De-identified and cleaned data were entered into NVivo (QSR International) [12] to facilitate organization into analytical themes. The data were analyzed using a structured and rigorous approach of thematic content analysis [13]. Two members of the research team (AC and SHT) independently coded each interview before discussing with a third researcher (HS) to reach consensus. The themes are illustrated verbatim quotes identified with the two following descriptors: (1) type of consultation (telemedicine only [TM] or telemedicine plus referral to specialist center) and (2) patient’s study number. Our findings are reported in accordance with the Consolidated Criteria for Reporting Qualitative Research [14].

### Ethics Approval

This study was approved by the National Healthcare Group Domain Specific Review Board (ethics approval 2018/01112).

## Results

### Characteristics of Patients

A total of 21 interviews were conducted between March and July 2019. The participants’ age ranged from 22 to 72 years, and 65% (13/21) were male. These patients presented with rashes (11, 52%), pigmented lesions (4, 19%), itching (3, 14%), and dry skin (2, 10%). Moreover, 7 (33%) patients were referred to the National Skin Centre after their telemedicine consultation. Three major themes emerged from the transcripts: positive perceptions of teledermatology, concerns about teledermatology, and suggestions for improving the patient’s teledermatology journey.

### Patients’ Positive Perceptions of Teledermatology

#### Convenience

The patients generally found the teledermatology service convenient, reducing the need to travel elsewhere for a second opinion and minimizing their transport costs and loss of earnings.

It’s good for people who are working. They don’t have the time to go down and then they get the assurance, they get the results immediately.

TM 32

…you also have work schedule to conflict. And then sometimes, you know, you have better things to do.

TM 25

It’s like, it can be done over here, rather than going up to the skin centre and you have to spend most of the day at the skin centre. I’ve been there before and have to wait there quite a long time…saves time travelling…

TM 33

The convenience of teledermatology was recognized as being particularly beneficial for those with mobility problems.

It’s good for elderly also…Cause there’s no need to travel all the way there [National Skin Centre], like disabled, all these...

TM 30

#### Care in a Familiar Health Care Setting

Some users commented on their preference to be managed in a familiar health care environment rather than being challenged by navigating somewhere unfamiliar.

…for those, like for me, for the first time to go to the kind of new places [National Skin Centre] I need to, ah, google for the location...And go there, don’t know how, the way, the operation line, register, everything...

TM 9

#### Timely Consultation

Some skin conditions are intermittent. While it is relatively easy for patients to get a same day consultation in the polyclinic when they are symptomatic, there is no guarantee that these signs will persist or recur for an outpatient appointment days or weeks later.

…all the rashes, all the symptoms...they’re gone, during my appointment time…So it’s [teledermatology consultation] instant, can show to the specialist, my symptom, my sickness, everything, there on the spot...

TM 9

On occasions, because of diagnostic uncertainty or the severity of the skin issue, the teledermatology consultation resulted in an immediate referral to the National Skin Centre.

...my situation is quite serious, then it’s good ...they take a picture …then I can come to the Skin Centre to do all the things …. it’s fast.

TM plus referral to specialist center 45

#### Expert Involvement

The patients felt that receiving a medical opinion from a dermatologist was always preferable because of their expert knowledge about skin disorders.

But then, knowledge-wise, probably the skin doctor would be more knowledgeable about it…. It's more reliable…

TM 31

[Prefer] specialist to see my skin. [Family physician] may not be as trained as specialists.

TM 27

Feeling their management was informed by a specialist rather than a generalist, the patients spoke of the care plan in terms of being “reliable,” “right,” or “correct.”

…give the right advice, and then the right medicine.

TM 23

…the correct diagnosis, the correct medication is issued, and then my skin is better. The psoriasis is suppressed for now.

TM 25

Such comments about the relative status of the generalist and specialist were often balanced by complements about the polyclinic staff’s professionalism when arranging the teledermatology consultation.

...our doctors here [in the polyclinic] are very proficient… very proficient. They would know what the angle to take [of the photos for teledermatology]

TM 32

#### Reassurance

The very quick availability of a specialist’s clinical assessment, diagnosis, and management were reassuring to patients; they described how their anxieties were addressed and how they experienced a sense of relief.

…the telederm [teledermatology consultation] helped reduce that anxiety and the worry about the skin condition being contagious.

TM 35

…gives me the reassurance, because they can follow up on the spot instead of having to physically wait for like, maybe a few months for follow-up to see a real specialist.

TM 41

Then I get the results immediately … they give me the assurance there’s nothing sinister … I feel so happy.… So, it’s very calming effect.

TM 32

#### Better Prepared for Their Outpatient Appointment

Not all problems could be resolved by a teledermatology consultation, and some patients were thus referred for an outpatient consultation, diagnostic tests, and treatment at the national specialist center. Rather than resenting telemedicine as an unnecessary and additional step in the referral pathway to dermatology, some patients considered it helpful, describing how the dermatologist would already be familiar with their case.

…when I go to a skin centre, they already have my records…instead of like, when I go there, they will start from scratch or they didn’t know what happened to me. But at least now, they have also my picture …And they have a more, like, the background profile…So, when I go there, maybe, it’s a bit faster.

TM plus referral to specialist center 38

#### Speed of Specialist Response

Although the teledermatology service is store-and-forward rather than a video consultation, many patients valued the short interval between presenting for their polyclinic appointment and receiving advice from a dermatologist later that day. Interestingly, although the service was asynchronous, some used descriptors such as “instant” or “immediate.”

…this [teledermatology] is quite unique, and quite good, because this feedback is immediate. So, you don’t have to delay. So at least they [doctors] have first-hand information. It eases the patients’ anxiety.

TM plus referral to specialist center 14

It is fast, and I can see on the same day. You get the instant result …

TM 32

…I liked it that the advice was given immediately …Very timely.

TM plus referral to specialist center 38

#### Consultation With a Specialist Without the Cost

Unlike the health care system in many socialist nations, Singaporeans cannot walk into a health care facility and receive treatment for free. Instead, Singapore imposes user fees, a policy designed to reduce inappropriate and unnecessary use of medical services. Therefore, while an outpatient appointment with a dermatologist would normally incur some fee, access via telemedicine to a specialist opinion incurred no costs for the patient beyond that of consultation with a primary care practitioner.

Cost wise… [I] only pay for consultation to see doctor here [National Healthcare Group Polyclinics], then the specialist, no need to pay.

TM 30

### Patients’ Concerns About the Teledermatology Service

#### Waiting Time

While patients valued having a dermatologist’s opinion and a definitive care plan on the same day as their visit to the polyclinic, there were conflicting views on the waiting times. We saw above how some service users commented on the immediacy of the feedback, but others expressed discontent about the time they waited before the dermatologist responded. Not only was the duration perceived as inconveniently long, but there was also concern about the uncertainty and unpredictability of the waiting time.

…at the polyclinic, I was told to wait for, like, maybe, like, two hours…I understand…the doctors might be busy…But the waiting time is probably one of the hindrance…

TM 31

For some patients, having waited for advice from the teledermatologist, they found that a trip to the specialist center was still going to be necessary. These patients often expressed surprise, indicating it their frustration about this unexpected outcome.

It seems like a waste of time…come to a big round…we are referred to the specialist, we are going through the same old thing, we wait for weeks for appointment, and we, doesn’t [sic] know what happened to us.

TM plus referral to specialist center 40

#### Apparent Unsophistication of the Equipment Used

Some patients commented on the simplicity of the photographic equipment used and wondered if the pictures had sufficient clarity for an accurate diagnosis.

They use a camera…like normal camera only, …. cannot zoom, I don’t think the quality of camera is good, I don’t think so.

TM plus referral to specialist center 45

…it wasn't a special camera, where they can adjust the light or pixel… I think it was his personal phone or it was a government phone…

TM plus referral to specialist center 10

…[general practitioner (GP)] could … take multiple views, multiple shots, instead of two pictures…I think one picture with light, one picture under bright light, maybe with, under bright light skin appear different…Bit more information, bit more input for the specialist to see, so he can do a better picture… more accurate diagnosis.

TM 25

Comments about equipment were intertwined with issues of security, allayed in part by the consent form.

Even though it’s just using a phone, it’s not a very professional way, but at least there’s this form [consent form], whereby you know that it’s still safe and you can daringly allow them to take the picture.

TM 33

The patients wanted more information about what personal details were being shared between the 2 institutions. Data security was perhaps in the forefront of their minds as the interviews were conducted soon after an incident in Singapore where some sensitive information had been mismanaged and other data misappropriated by computer hackers.

…let the patient know …. know what was shared with the skin centre.

TM 17

The sending of photographic images was not considered as risky as information transmitted in text format. The photos were considered generally to maintain anonymity, as illustrated by the following quote:

I mean they will actually focus on your areas that was affected and try to take a clear picture…And they will try to avoid your face, features…

TM 35

#### Unavailability of the Recommended Medication in the Polyclinic

As a primary care medical facility, the polyclinic dispensaries did not always have the medication recommended by the dermatologist. The patients then had to go elsewhere or wait for their medications to be delivered to the polyclinic pharmacy.

Of course, they [the specialist center] have lots, lots of creams, because they are looking after the skin, so they got whole range of, of treatment. Sometimes, some of the creams [the polyclinic] may not have.

TM 32

### Patients’ Suggestions for Improving Their Teledermatology Journey

The interviewees recognized that the start to finish time for teledermatology was much shorter than a conventional polyclinic referral to the specialist center and outpatient attendance, which could be many weeks later. However, for some patients, the time spent at the polyclinic was felt to be unnecessarily long and unpredictable and an aspect of the service needing refinement. Delays could happen at several points within the process, including the internal referral from the attending clinician to the “Derm Champ” to initiate the teledermatology process, the setting up of the camera, and the time waiting for a response from the dermatologist. Patients who had experienced delays for the camera to be set up wondered if there could be a dedicated facility to minimize the time spent preparing for the teledermatology referral.

…things like the camera, the equipment, everything is ready when the patient comes in. Take photo immediately, then just upload.

TM 9

The interval between referral and response was not predictable as it depended on the availability of the receiving dermatologist who fitted the teleconsultations in between their other clinical commitments. The resultant undefined waiting time when having scheduled a standard polyclinic appointment was not always convenient for the patient. Their suggestions for reducing the amount of waiting time needed to be spent in the polyclinic included allowing patients to leave the polyclinic after their clinical data had been transmitted to the dermatologist, and to be contacted later in the day with details of the management plan proposed.

…maybe I’m able to receive message, or phone mobile message, by phone, then it’s okay, maybe, then faster.

TM 17

Such suggestions about the adoption of more technology into the teledermatology process was at variance with the views of others who wanted greater opportunity to debrief and discuss with the referring GP about the recommended management plan. Such discussions were particularly valued when the diagnosis had implications for work, lifestyle, or the well-being of others.

…explain better on the care plan. Yeah, because it’s a suspected diagnosis, it’s not like a…confirmed diagnosis. So, I’m very scared because scabies is contagious. I have my kid at home, and my husband is sleeping with me…When I left the clinic, I was, I was worried.

TM 28

The validity of the overall positive feedback was also evidenced by the many requests to expand teledermatology. The patients described their surprise on encountering this facility and challenged if the level of awareness of the service was sufficient.

How many patients know about this? I think [a] publicity programme. I don’t know if the public is aware of this.

TM plus referral to specialist center 29

Those patients with good experiences felt that the teledermatology service should not only be promoted within the polyclinics, but that access should be also extended to those patients who attend a private GP for their primary health care. One respondent envisaged the development of a mobile teledermatology service to facilitate solo GP clinics using the service.

…think of is like blood test, X-ray; if there is a mobile service, people may just attend to it… More accessible, not just at the Polyclinic.

TM 14

## Discussion

### Principal Findings

This study explored the experiences of patients using a teledermatology service linking a public polyclinic with a specialist dermatology service in Singapore. The patients found the teledermatology service convenient, saving them time and expense. It liberated them from the stresses incurred when making an in-person visit to a specialist facility. They valued the confidence and reassurance they had from the specialist’s input to the management plan. The patients expressed concerns related to the security of their personal data as it was transferred between institutions; the unpredictability of the time spent waiting; the fact that the virtual telemedicine consultation may not necessarily dispense with the need for an in-person visit to the specialist center; the apparent unsophistication of the photographic equipment; the lack of the recommended medication within the polyclinic; and the lack of adequate closure of the consultation. The patients were keen to see the service advertised and made available beyond the polyclinic.

Gradually, health care is moving away from the traditional, rather paternalistic health service that “does things for its patients” and toward one that is more patient-led in both design and organization [15]. Addressing patient’s experiences enables the development of more patient-focused care, which in turn improves satisfaction and health outcomes [16]. Using the in-depth qualitative interviews, we were able to gain insight into the experiences and views of adult patients; such information may not be apparent in quantitative, fixed-response patient satisfaction surveys [17]. There were several aspects of the patients’ telemedicine journey that they found inconvenient. These included uncertainty about the total duration of a telemedicine consultation and the unavailability of recommended medication from the polyclinic pharmacy. The lack of clarity about the total amount of time needed to complete the consultation and obtain a management plan was in part due to the use of “store-and-forward” telemedicine. This asynchrony was inevitable as the dermatologist on duty had other clinical duties running in parallel with their responsibilities for fielding the telemedicine calls. The multitasking of the recipient specialist will always be a workforce planning challenge if the referral institutions are not generating sufficient cases for the full-time attention of the clinician in receipt of referrals. The patients’ satisfaction with the concept of virtual consultation was apparent when they spoke of a desire to see widening access to the telemedicine service beyond the polyclinic. The patients suggested that the service could be expanded to include private general practices and that the public should be made aware of its availability. Certainly, the expansion of the teledermatology service to additional sites and the introduction of an efficient electronic queuing system could justify the allocation of dedicated staff and more predictable turnaround times for patients.

### Strengths and Weaknesses

This paper adds to the small number of qualitative studies [18] of teledermatology to be found among a rapidly growing quantitative literature on diagnostic accuracy [19], cost-effectiveness [20], and patient outcomes [21]. The advantage of a narrative approach is that it produces rich data enabling a better understanding of the patient’s journey and the way they understand and interpret their experiences. For example, some interviewees interpreted the clinician putting on gloves before examining their skin as reticence engaging with them, rather than as a hygiene measure [22]. Such patient concerns illustrate how things that may be entirely reasonable to health care professionals may be challenging to a layperson if not explained.

There was diversity in the patient’s responses; for example, some perceived the teledermatology consultation service as quick while others described it as a protracted experience. As service providers, we may see the organization of teledermatology as standardized and streamlined, failing to recognize that the journeys of individual patients are quite diverse, with different trajectories (eg, the involvement of 1 or 2 primary care doctors), different durations (eg, waiting times and delays), and different outcomes (eg, a management plan that can be implemented in primary care or an outpatient visit to the specialist center).

Being more attentive in our interviews to the patient’s anticipated configuration of their journey and comparing patient expectations with the reality would have helped us develop a deeper understanding of incongruity. Perhaps those reporting a quick service were taking as their baseline previous experiences of referral to an outpatient clinic, whereas those who perceived it as slow were using the routine polyclinic waiting time of less than 10 minutes as their baseline [9]. A future qualitative study using purposive sampling of patients who had experienced the different patient pathways will help us explore these complexities further.

### Conclusions

Recognizing that patients value telemedicine for its convenience and being less demanding on time and money, Duffy and Lee [23] recently posed the question whether in-person visits should become the second, third, or even last options for meeting patients’ needs? This proposal challenges the traditional way of providing care; even though telemedicine has been available in many countries for more than 20 years, its incorporation into patient care has been patchy and often confined to remote areas, or where there is a paucity of appropriate expertise. In countries where remuneration is fee-for-service, the adoption of telemedicine has been complicated by disputes over the disparities in pay for telemedicine versus in-person care (eg, in the United States, there is parity in only one-fifth of the states [24]). With the COVID-19 pandemic in Singapore, we are seeing telemedicine being used more widely to reduce travel and enable safe distancing [25,26]. Perhaps this pandemic will provide the catalyst for practice redesign, with in-person health care becoming the second rather than the first option for patient care.

## Appendix

Multimedia Appendix 1 Topic guide for semistructured interviews.

## Abbreviations

GP: general practitioner
TM: telemedicine
