# Supplementary material for: Patients’ Experiences of Telemedicine for Their Skin Problems: Qualitative Study
Source: JMIR Dermatol. 2022 Feb 22;5(1):e24956. doi: 10.2196/24956 (PMC10334905; doi:10.2196/24956)
Supplement: Multimedia Appendix 1 [file derma_v5i1e24956_app1.docx]

**Appendix 1: Topic guide for semi-structured interviews**

**Protocol Title:**

An Evaluation of Patients’ and Health Professionals’ Experiences of a Teledermatology Service in Singapore

**TOPIC GUIDE**

**Introduction**

We are interested in exploring patients’ and health professionals’ perceptions of the teledermatology service in Singapore, and how this service can be improved to bring better quality of care to patients. We would like to invite you to participate in an approximately 30 to 40 minutes interview, to share your views and experience of the teledermatology services. The interview will be recorded but your views are anonymous for the purpose of transcription, analysis and publication. All information collected (including the digital recording) will be stored securely and destroyed after 10 years.

**Are you agreeable for recording of the interview session?**   Yes /  No

| **Gender:** | M / F | | | |
| --- | --- | --- | --- | --- |
| **Age:** |  |  | years old |  |

| **Ethnic:** | | Chinese  Malay  Indian  Others | |
| --- | --- | --- | --- |
| **Date of teledermatology consultation:** |  | |  |

**Let’s begin by sharing with me…**

1. What were your initial thoughts/reactions when your family doctor suggested using the teledermatology service (instead of the usual specialist referral method)?
2. What were the problems you have been experiencing with your skin?
3. What did you like about the teledermatology service?
4. Which of these points (mentioned in Q3) was the most important to you?
5. What did you not like about the teledermatology service? Please explain/tell me more.
6. In what ways has this teledermatology service helped you cope and manage with your skin problem?

*Prompt: Presenting symptom, diagnosis, reason for consulting, benefits, follow-up appointments*

1. Prior to using the teledermatology service, had you heard of any Telemedicine Health Services?
2. Would you recommend the teledermatology service to your friends and relatives? What is the reason for your response? Tell me more about it.

**Last but not least, for the future**

1. How can the teledermatology service be improved?

*Prompt only when needed: consultation process, referral process*

**Thank you for your time and effort in participating in this interview.**
